# Who would be targeted by increasing the legal age of sale of cigarettes from 18 to 21? A cross‐sectional study exploring the number and characteristics of smokers in England

**DOI:** 10.1111/add.15421

**Published:** 2021-02-10

**Authors:** Emma Beard, Jamie Brown, Sarah E. Jackson, Robert West, Will Anderson, Deborah Arnott, Lion Shahab

**Affiliations:** ^1^ Department of Behavioural Science and Health University College London London UK; ^2^ Action on Smoking and Health (ASH) London UK; ^3^ Division of Epidemiology and Public Health, School of Medicine University of Nottingham

**Keywords:** Age of sale, England, policy, smoking, tobacco control, youth

## Abstract

**Aims:**

To establish the number of smokers in England who would be targeted by increasing the age of sale of cigarettes from 18 to 21 years and to assess the smoking and socio‐demographic profile of those smokers.

**Design and setting:**

Nationally representative cross‐sectional survey of adults in England conducted between January 2009 and July 2019.

**Participants:**

A total of 219 720 adults.

**Measurements:**

All participants reported their current smoking status and socio‐demographic characteristics (i.e. age, gender, home ownership, social grade and ethnicity). Smokers reported motivation to quit, urges to smoke and the Heaviness of Smoking Index (HIS). Weighted prevalence statistics were calculated. Multinomial regression and logistic regression were used to assess differences in smoking characteristics among smokers and socio‐demographic characteristics relative to non‐smokers.

**Findings:**

The prevalence of smoking between January 2009 and July 2019 was highest among those aged 21–30. In 2019, 15.6% [95% confidence interval (CI) = 12.8–18.8%] of 18–20‐year‐olds reported smoking, which is estimated to represent 364 000 individuals in England. Relative to smokers aged 18–20, older smokers (aged 21+) had a higher motivation to quit smoking [odds ratios (ORs) = 1.40–1.45 range] and higher nicotine dependency as measured by urges to smoke (ORs = 1.06–1.24 range) and HSI (ORs = 1.05–2.85 range). Compared with non‐smokers aged 18–20, smokers in this age group had lower odds of being female (OR = 0.89) and higher odds of being of white ethnicity (OR = 2.78) and from social grades C1–E (lower social grades) compared with AB (higher social grades) (OR = 1.19–1.83 range).

**Conclusion:**

Increasing the age of sale of cigarettes to 21 years in England would currently target approximately 364 000 lower dependent smokers from more disadvantaged backgrounds aged 18–20, who have less motivation to quit.

## Introduction

Most tobacco use starts during adolescence, and the vast majority of people who are regular smokers during these years become addicted to tobacco [1, 2, 3]. There is a variety of reasons why young people take up smoking. Some think it is fashionable, while others are influenced by social pressure and smoking among friends and family members [4, 5]. Impulsivity and related characteristics, low socio‐economic status and poor mental health, such as experiencing depression or stress, can also increase the risk of starting to smoke [6, 7, 8, 9]. Heritability is estimated to account for 60% of the variance in smoking initiation and between 55% and 69% in smoking persistence [10].

Numerous evidence‐based strategies are effective in preventing tobacco use in young people: mass media campaigns, taxation, advertisement restrictions, smoke‐free policies and access restrictions [11]. In relation to the last of these, studies have demonstrated the long‐ and short‐term efficacy of the increase in the legal age of sale of cigarettes in England from 16 to 18 years on 1 October 2007. There was a greater immediate fall in smoking prevalence in 16–17‐year‐olds following the increase in age of sale than in older age groups and a greater long‐term decline in ever smoking among those aged 16–17 compared with those aged 18–24 [12, 13, 14, 15].

Recently, there have been calls to raise the age of sale further to 21 years in England [16]. The logic behind this is that extending access restrictions to 18–20‐year‐olds makes the process of obtaining cigarettes, both through social sources and purchase, more difficult, not just for 18–20‐year‐olds but also for younger people. A decrease in peer use by older teenagers may also make smoking less socially desirable to younger teenagers [17]. As a result, it could decrease the number of young people trying smoking, and the proportion of young smokers who go on to become regular adult smokers. Studies have shown public support for this policy, and evidence for its efficacy is emerging following its implementation in 16 US states, including Hawaii [16, 18, 19, 20, 21, 22].

The possible public health benefits of increasing the legal age of sale to 21 were summarized in a report by the Institute of Medicine. This includes the direct effects of averting premature deaths and life‐years lost, as well as reducing the effects of second‐hand smoking, which contributes to low birth weights and sudden infant death syndrome [23].

In making decisions about whether or not to raise the age of sale to 21 years, it is important for policymakers to understand the size and profile of the population of adult smokers in England aged 18–20 years who would be affected. It is also necessary to assess whether this increase in age of sale if it were to be implemented is likely to disproportionately affect any specific socio‐demographic groups and specific profile of smokers. Should the increase in legal age of sale be implemented, this knowledge will help to inform ways in which to educate the public and plan for the provision of necessary smoking cessation support. It will also elucidate whether an increase in the legal of age of sale could reduce social inequalities in health if, for example, those from lower socio‐economic backgrounds are more likely to be targeted.

The objectives of this study were therefore:

1. To report on the prevalence of smoking in England among those aged 18–20 compared with other age groups and thereby establish the number of adult smokers who would be currently targeted (by definition, it would be illegal to sell tobacco to those aged 18–21) by the increase in age of sale.
2. To assess whether the increase in age of sale would disproportionately affect specific groups of smokers by establishing whether the smoking profile of 18–20‐year old smokers is different to smokers in older age groups.
3. To assess whether the increase in age of sale would disproportionately affect specific socio‐demographic groups by establishing whether the ethnic, gender and socio‐economic profile of 18–20‐year old smokers is different to non‐smokers.

## Methods

### Design

Data were used from the Smoking Toolkit Study (STS; www.smokingengland.info), an ongoing survey of smoking behaviour in England that began in November 2006 [please contact the lead author for access to the STS and R syntax]. The STS involves monthly cross‐sectional household computer‐assisted interviews, conducted by Ipsos Mori of approximately 1700 adults aged 16+ in England. The baseline survey uses a type of random location sampling, which is a hybrid between random probability and simple quota sampling. Participants from the STS appear to be representative of the population in England, having similar socio‐demographic composition and smoking characteristics to large national surveys based on probability samples such as the Health Survey for England [12]. Estimates of cigarette consumption from the Smoking Toolkit Study and sales data are closely aligned [24].

For the present analysis, we used data collected between January 2009 (the first wave to assess motivation to quit in a full‐sized sample) and July 2019 (the latest wave of data available at the time of analysis). Between these time‐points the age of sale has remained constant at 18. Use of data over this 10‐year period allows an adequate sample size to assess the current impact an increase in age of sale would have in terms of smoking and demographic characteristics. We have shown previously that most of the characteristics reported here are time‐invariant [25], thus aggregating data across a decade will provide a more accurate overall estimate.

### Measures

Smoking status was assessed by asking participants: ‘Which of the following best applies to you? (a) I smoke cigarettes (including hand‐rolled) every day; (b) I smoke cigarettes (including hand‐rolled), but not every day; (c) I do not smoke cigarettes at all, but I do smoke tobacco of some kind (e.g. pipe or cigar); (d) I have stopped smoking completely in the last year; (e) I stopped smoking completely more than a year ago; (f) I have never been a smoker (i.e. smoked for a year or more)’. Smokers were defined as those who answered (a), (b) or (c).

Data were collected on smokers’ age (categorized as 18–20, 21–24, 25–30, 31+), gender, ethnicity, social grade and housing tenure. Social grade was measured using the British National Readership Survey (NRS) Social‐Grade Classification Tool [26]: AB (higher managerial, administrative or professional), C1 (supervisory or clerical and junior managerial, administrative or professional), C2 (skilled manual workers), D (semi‐skilled and unskilled manual workers) and E (casual or lowest grade workers, pensioners and others who depend upon the welfare state for their income). Housing tenure was measured by asking participants which of the following applied to them: (1) bought on a mortgage; (2) owned outright; (3) rented from local authority; (4) rented from private landlord; (5) belongs to housing association; (5) other. This was dichotomized to distinguish between those who did versus did not own their own home (i.e. number 1 and 2 versus others). This measure has been identified as a strong socio‐economic predictor of smoking status in England [27].

Motivation to quit was assessed using the Motivation to Stop Scale (MTSS) [28]. Current smokers were asked: ‘Which of the following describes you? (1) I don't want to stop smoking; (2) I think I should stop smoking but don't really want to; (3) I want to stop smoking but haven't thought about when; (4) I really want to stop smoking but I don't know when I will; (5) I want to stop smoking and hope to soon; (6) I really want to stop smoking and intend to in the next 3 months; (7) I really want to stop smoking and intend to in the next month’. High motivation to quit was derived by dichotomizing into those who wanted and intended to quit in < 3 months (i.e. number 6 and 7 versus others). This categorization has been shown to be related to future quitting activity [29].

We used two measures of cigarette dependence: Heaviness of Smoking Index (HSI) and Strength of Urges to Smoke (SUTS). The HSI was calculated from the number of cigarettes smoked per day (1–10 = 0, 11–20 = 1, 21–30 = 2, 31+ = 3) and the time to first cigarette after waking (≤ 5 = 3, 6–30 = 2, 31–60 = 1 and 61+ minutes = 0). The HSI summary score ranges from 0 (lowest) to 6 (highest level of dependence) with scores of 2–4 and 5–6 generally seen to represent medium and high dependency, respectively [30]. SUTS was measured by asking: ‘In general, how strong have the urges to smoke [in the past 24 hours] been?’ slight (1), moderate (2), strong (3), very strong (4), extremely strong (5). This question was coded ‘0’ for smokers who responded ‘not at all’ to a previous question asking: ‘How much of the time have you felt the urge to smoke in the past 24 hours?’ [31].

### Analysis

The analysis plan was pre‐registered on the Open Science Framework (https://osf.io/s8k69). An amendment was made to the analysis plan in August 2019. We included housing tenure as a correlate following evidence that it was strongly and independently associated with smoking status across age groups [27]. We also split the older age group into three categories: 21–24, 25–30 and 31+ to provide a more detailed comparison of the differences in characteristics between smokers aged 18–20 and older smokers. Finally, analyses were restricted to post‐January 2009. Questions on motivation to quit were not introduced until November 2008, and estimates based on the last 2 months of 2008 were considered likely to be inaccurate due to small sample sizes.

All analyses were conducted in R Studio. For objective 1, smoking prevalence was reported for those aged 18–20 and those aged 21–24, 25–30 and 31+, overall and as a function of year. Prevalence estimates were weighted using a rim (marginal) weighting technique [32]. This involves an iterative sequence of weighting adjustments whereby separate nationally representative target profiles are set (for age, social grade, region, tenure, ethnicity and working status within gender). This process is then repeated until all variables match the specified targets. A simple trend analysis then calculated the absolute and relative change in smoking among age groups. An estimate of the number of those aged 18–20 who would currently be targeted by an increase in the legal age of sale of cigarettes to 21 was then calculated using the Office for National Statistics census estimates of population size for adults aged 18+ [33] and prevalence statistics from 2019.

For objective 2, multinomial logistic regression was used to assess whether there were any differences between smokers in the four age groups as a function of smoking characteristics, with smokers aged 18–20 as the reference category. Univariable models and multivariable models are reported. All models adjusted for time (year of survey) in the analysis, while the multivariable models also adjusted for socio‐demographic characteristics and all other smoking variables in the model (i.e. motivation to quit, HSI and urges to smoke). Data were used from 2009 until 2019.

For objective 3, logistic regression was used to assess whether there were any differences between smokers and non‐smokers as a function of socio‐demographic characteristics, across by age group (i.e. separate models for each age group comparing smokers and non‐smokers). Univariable models and multivariable models are reported. All models adjusted for time (year of survey) in the analysis, while the multivariable models also adjusted for all other variables in the model. Data were used from 2009 until 2019.

Sensitivity analyses were run for objectives 2 and 3 restricting the analysis to 2019 following reviewer comments. Findings were broadly similar to those presented in the paper and are given in the Supporting information.

## Results

### Sample size

Data were collected on 219 720 participants aged 18+ between January 2009 and July 2019. Of these, 12 133 [5.53%, 95% confidence interval (CI) = 5.43–5.63%] were aged 18–20, 16 396 (7.47%, 95% CI = 7.58–7.36%) were aged 21–24, 22 164 (10.10%, 95% CI = 9.97 to 10.23%) were aged 25–30 and 168 742 (76.90%, 95% CI = 76.72–77.07%) were aged 31+.

### Prevalence of smoking

In 2019, 15.6% (95% CI = 12.8–18.8%) of those aged 18–20 were smokers, which accounts for 364 000 individuals in England based on 2019 mid‐year census estimates. On the basis of the average prevalence from 2009 and 2019 of 23.8%, this would account for 54 007 692 individuals. Smoking prevalence in 2019 among those aged 21–24, 25–30 and 31+ was 20.0% (17.4–22.8%), 24.3% (21.9–26.9%) and 14.2% (13.5–14.9%), respectively.

Table 1 shows that prevalence of smoking over time has been consistently highest among those aged 21–30, with the greatest relative change over time among those aged 18–20 (a decline of 46.6% between 2009 and 2019).

**Table 1:** Prevalence of smoking as a function of year and age group.

Year	All age groups				Aged 18–20				Aged 21–24				Aged 25–30					Aged 31+		
	%	Lower 95% CI	% (95% CI) *n*	*n*	%	Lower 95% CI	Upper 95% CI	*n*	%	Lower 95% CI	Upper 95% CI	*n*	%	Lower 95% CI	Upper 95% CI	*n*	%	Lower 95% CI	Upper 95% CI	*n*
2009	22.2	21.7	22.8	20 616	29.2	26.7	31.8	1283	31.9	29.5	34.3	1479	29.0	27.0	31.0	2006	19.9	19.3	20.5	15 848
2010	22.0	21.5	22.6	24 154	27.8	25.5	30.2	1393	31.2	29.1	33.4	1814	29.3	27.5	31.2	2389	19.8	19.2	20.4	18 558
2011	21.4	20.8	21.9	21 146	25.7	23.3	28.3	1172	27.6	25.4	29.9	1550	28.1	26.3	30.0	2207	19.5	18.9	20.1	16 218
2012	20.7	20.2	21.3	20 721	24.6	22.2	27.2	1165	26.4	24.2	28.6	1597	25.4	23.5	27.3	2078	19.2	18.6	19.8	15 881
2013	19.8	19.3	20.4	21 588	24.3	21.9	26.9	1145	28.1	25.9	30.3	1624	26.9	25.0	28.8	2194	17.8	17.2	18.4	16 625
2014	19.1	18.6	19.7	19 701	22.2	19.8	24.9	1060	27.6	25.4	30.0	1474	26.7	24.8	28.7	1982	17.1	16.5	17.7	15 184
2015	19.3	18.8	19.9	19 542	24.8	22.2	27.7	981	27.5	25.3	29.8	1528	27.4	25.5	29.4	1991	17.1	16.5	17.7	15 042
2016	18.6	18.1	19.2	19 924	22.5	20.1	25.1	1098	26.8	24.5	29.1	1464	24.4	22.6	26.4	1990	16.8	16.2	17.4	15 372
2017	17.7	17.2	18.3	19 910	20.3	18.0	22.9	1109	27.1	24.9	29.5	1455	26.4	24.5	28.3	2048	15.5	14.9	16.1	15 298
2018	17.8	17.3	18.3	20 289	18.8	16.6	21.3	1125	25.0	22.8	27.2	1529	26.0	24.1	27.9	2087	15.9	15.4	16.5	15 548
2019^*^	15.7	15.0	16.4	11 675	15.6	12.8	18.8	593	20.0	17.4	22.8	864	24.3	21.9	26.9	1171	14.2	13.5	14.9	9049
All years	19.7	19.5	19.9	219 267	23.8	23.0	24.6	12 124	27.5	26.9	28.2	16 377	26.9	26.3	27.5	22 142	17.7	17.5	17.9	168 624
Absolute change from 2009 to 2019 (ppt)	6.5				13.6				11.9				4.7				5.6			
Relative change from 2009 to 2019 (%)	29.3				46.6				37.3				16.2				28.1			
OR (95% CI) *P* for 2019					Ref				1.25 (1.19–1.32) < 0.001				1.20 (1.14–1.27) < 0.001				0.71 (0.68–0.74) < 0.001			

^*^ Only half‐year data. OR= odds ratio; CI = confidence interval.

### Associations between smoking characteristics and age

Table 2 shows the smoking characteristics of smokers as a function of the age groups of interest. Table 3 shows the results of the regression analyses. The fully adjusted model revealed significant differences in all measured characteristics by age group. Relative to older smokers (all 21+ age groups), smokers aged 18–20 had lower nicotine dependency (as measured by the HSI and SUTS), but a lower odds of reporting high motivation to quit smoking. The odds of reporting high motivation to quit smoking were almost 50% higher among those aged 21+.

**Table 2:** Socio‐demographic and smoking characteristics as a function of age and smoking status.

	Smokers										Non‐smokers									
	Overall		Aged 18–20		Aged 21–24		Aged 25–30		Aged 31+		Overall		Aged 18–20		Aged 21–24		Aged 25–30		Aged 31+	
	*N*	%	*n*	%	*n*	%	*n*	%	*n*	%	*n*	%	*n*	%	*n*	%	*n*	%	*n*	%
Female	21 469	47.66	1329	45.61	2148	46.32	2854	49.16	15 138	47.77	90 834	51.92	4147	45.27	5607	48.19	8468	55.90	72 612	52.24
Social grade	5040	11.30	224	7.69	308	6.64	548	9.44	3960	12.67	33 342	24.07	1134	12.28	1437	12.35	2709	17.88	28 062	27.38
AB (highest)	10 945	24.54	946	32.46	1366	29.46	1380	23.77	7253	23.21	54 683	31.26	3778	41.24	4685	40.27	5321	35.12	40 899	29.43
C1 C2	9985	22.38	561	19.25	965	20.81	1355	23.34	7104	22.73	33 704	19.26	1651	18.02	2307	19.83	3249	21.45	26 497	19.06
D	8609	19.30	608	20.86	1022	22.04	1317	22.68	5662	18.12	23 143	13.23	1529	16.69	1956	16.81	2507	16.55	17 151	12.34
E (lowest)	10 032	22.49	575	19.73	976	21.05	1206	20.77	7275	23.28	20 064	11.47	1068	11.66	1249	10.74	1363	9.00	16 384	11.79
Owns home	9113	38.65	423	26.74	536	20.16	648	22.01	7506	45.79	69 107	66.45	2227	37.14	2371	31.95	3235	38.59	61 274	74.55
White	20 918	89.09	1367	86.63	2292	86.36	2516	85.69	14 743	90.38	87 154	84.16	4300	71.93	5456	73.80	5917	70.87	71 481	87.35
Motivation to quit	8021	17.74	493	16.88	855	18.35	1163	19.99	5510	17.32										
HSI low	19 815	38.06	1597	50.65	2463	29.26	3034	47.73	12 721	37.27										
(ref) mediu	2673	54.96	1480	46.94	2413	48.26	3168	49.84	19 676	57.64										
m high	2091	4.30	76	2.41	124	2.48	154	2.42	1737	5.09										
			M	SD	M	SD	M	SD	M	SD										
Urges to smoke	1.9	1.1	1.81	1.13	1.83	1.10	1.90	1.08	2.06	1.09										

SD = standard deviation; M = mean.

**Table 3:** Association between age and smoking characteristics (reference 18‐20 year‐olds).

	Model 1: adjusted for year of survey									Model 2: fully adjusted								
	Aged 21–24			Aged 25–30			Aged 31+			Aged 21–24			Aged 25–30			Aged 31+		
	OR	95% CI	*P*	OR	95% CI	*P*	OR	95% CI	*P*	OR	95% CI	*P*	OR	95% CI	*P*	OR	95% CI	*P*
Motivation to quit	1.13	1.07–1.19	< 0.001	1.22	1.16–1.29	< 0.001	1.03	0.99–1.07	0.138	1.45	1.35–1.57	< 0.001	1.49	1.38–1.61	< 0.001	1.40	1.32–1.49	< 0.001
HSI low (ref)	1.11	1.06–1.16	< 0.001	1.14	1.10–1.19	< 0.001	1.76	1.71–1.81	< 0.001	1.20	1.13–1.27	< 0.001	1.35	1.27–1.44	< 0.001	1.90	1.81–1.99	< 0.001
Medium high	1.19	1.18–1.19	< 0.001	1.21	1.21–1.21	< 0.001	3.31	3.31–3.32	< 0.001	1.05	1.05–1.06	< 0.001	1.11	1.11–1.12	< 0.001	2.85	2.84–2.87	< 0.001
Urges to smoke	1.04	1.00–1.09	0.066	1.09	1.05–1.14	< 0.001	1.27	1.22–1.31	< 0.001	1.06	1.00–1.12	0.053	1.14	1.07–1.20	< 0.001	1.24	1.18–1.30	< 0.001

HSI = Heaviness of Smoking Index; OR = odds ratio; CI = confidence interval.

All models adjusted for year of survey; model 2 also adjusted for socio‐demographic characteristics and all other variables in the model.

### Associations between socio‐demographic characteristics and smoking status across age groups

Table 2 shows the socio‐demographic characteristics of smokers and non‐smokers as a function of the age groups of interest. Table 4 shows the results of the regression analyses. When comparing smokers and non‐smokers aged 18–20, smokers had a significantly higher odds of being in lower social grades and lower odds of owning their own home. They also had higher odds of being of white ethnicity and lower odds of being female. Older age groups showed a similar pattern of results.

**Table 4:** Univariable and multivariable association between smoking status (non‐smoker reference) and socio‐demographic characteristics stratified by age.

	Aged 18–20			Aged 21–24			Aged 25–30			Aged 31+		
	OR	95% CI	*P*	OR	95% CI	*P*	OR	95% CI	*P*	OR	95% CI	*P*
Model 1												
Female	1.03	0.94–1.12	0.563	0.93	0.87–0.99	0.032	0.77	0.72–0.82	< 0.001	0.88	0.86–0.90	< 0.001
Social grade												
AB (ref)												
C1	1.29	1.10–1.52	0.002	1.42	1.24–1.63	< 0.001	1.29	1.16–1.44	< 0.001	1.75	1.68–1.83	< 0.001
C2	1.70	1.43–2.03	< 0.001	2.01	1.74–2.33	< 0.001	2.09	1.87–2.34	< 0.001	2.65	2.54–2.76	< 0.001
D	1.95	1.64–2.32	< 0.001	2.47	2.13–2.86	< 0.001	2.62	2.34–2.94	< 0.001	3.26	3.11–3.41	< 0.001
E	2.69	2.26–3.21	< 0.001	3.72	3.20–4.33	< 0.001	4.35	3.86–4.91	< 0.001	4.28	4.10–4.48	< 0.001
Owns home	0.61	0.54–0.69	< 0.001	0.52	0.47–0.58	< 0.001	0.45	0.41–0.50	< 0.001	0.28	0.27–0.29	< 0.001
White	2.62	2.24–3.08	< 0.001	2.36	2.09–2.68	< 0.001	2.57	2.29–2.89	< 0.001	1.37	1.30–1.46	< 0.001
Model 2												
Female	0.89	0.79–0.99	0.039	0.82	0.75–0.90	< 0.001	0.71	0.65–0.78	< 0.001	0.81	0.79–0.84	< 0.001
Social grade												
AB (ref)												
C1	1.19	0.96–1.50	0.117	1.29	1.07–1.55	0.008	1.12	0.97–1.31	0.131	1.58	1.50–1.68	< 0.001
C2	1.55	1.22–1.50	< 0.001	1.79	1.48–2.18	< 0.001	1.79	1.53–2.09	< 0.001	2.12	2.00–2.52	< 0.001
D	1.83	1.43–2.35	< 0.001	2.27	1.86–2.77	< 0.001	2.35	2.01–2.77	< 0.001	2.37	2.22–2.52	< 0.001
E	1.74	1.34–2.35	< 0.001	2.27	2.21–3.36	< 0.001	3.21	2.67–3.85	< 0.001	3.05	2.85–3.26	< 0.001
Owns home	0.66	0.57–0.75	< 0.001	0.60	0.53–0.67	< 0.001	0.53	0.48–0.59	< 0.001	0.33	0.32–0.35	< 0.001
White	2.78	2.37 to 3.27	<0.001	2.37	2.09 to 2.69	<0.001	2.79	2.48 to 3.14	<0.001	1.95	1.84 to 2.07	<0.001

All models adjusted for year of survey; model 2 also adjusted for all other variables in the model. OR = odds ratio; CI = confidence interval.

## Discussion

### Summary of findings

This study aimed to establish the number of adult smokers in England who would be currently targeted by increasing the age of sale of cigarettes from 18 to 21 years and to assess the demographic and smoking profile of those smokers. In 2019, close to 16% of people aged 18–20 reported that they smoked tobacco, which equates to approximately 364 000 young smokers in England. Smokers aged 18–20 had lower nicotine dependence relative to smokers in other age groups, but were less motivated to quit. Compared with non‐smokers of a similar age, those aged 18–20 were less likely to be female and more likely to be of white ethnicity and of lower socio‐economic status (as measured by housing tenure and social grade).

### Implications

Studies have shown public support for increasing the legal age of sale to 21 and evidence for its efficacy is emerging [16, 18, 19, 20]. This study adds to the literature by estimating that if the age of sale is increased from 18 to 21 the number of young adults who would be currently targeted in England would be ~364 000. This could save the NHS £691 million a year if all of these young adults are prevented from transitioning into long‐term smokers (£1900 per smoker) [34]. However, poor compliance with access laws is well documented [35]. Thus, for this to be achieved it will also be imperative that retailers are educated about the new legislation and it is adequately enforced, as well as further strengthening controls on access to illicit tobacco [36].

It should also be noted that those aged 18–20 have seen the greatest relative decline over time in smoking prevalence compared with other age groups. This should be considered when taking into account the cost‐effectiveness of any increase in the age of sale, as it may reflect a group which is already highly responsive. Some of this decline may be a consequence of a longer‐term reduction in uptake of smoking following the increase in legal age of sale to 18 in 2007 [15].

The lower motivation to quit among smokers aged 18–20 suggests that they would be less likely than other‐aged smokers to attempt to quit in the future without policy intervention, such as an increase in the age of sale. Motivation may be lower among those aged 18–20 due to fewer immediate health concerns or long‐term worries about, or discounting of, the cost of smoking [37]. They may also be less likely to identify as a smoker [38].

The lower dependency of those aged 18–20 is probably a function of the lower number of years since the initiation of smoking for the behaviour to become established. Dependence is closely associated with successful quitting once an attempt is initiated [39]. This dependence profile should reduce policymaker concern of excessive financial burden in terms of cessation treatment. Behavioural and pharmacological interventions have proven efficacy for young adults in clinical trials, but may be less efficacious than for the adult population due to poor adherence [3, 40, 41].

It has been suggested that mass media campaigns are one of the most effective strategies in preventing youth smoking, and these would also need to be an integral part of any change in the legal age of sale [42]. In England, tobacco mass media campaigns have been run as part of a national tobacco control programme. Spending was almost completely suspended in 2010 and then re‐introduced in 2011 at a much lower level. Previous studies have shown that such cuts were associated with a decreased use of smoking cessation support across age groups and reduced quit success rates [43, 44, 45]. Ensuring that these campaigns continue to be adequately funded is therefore important, particularly should the increase in age of sale be implemented.

These findings also suggest that any increase in the legal age of sale may reduce social inequalities in health if those of lower socio‐economic status are less likely to take up smoking or are more likely to quit with its implementation. Smoking prevalence is generally higher among more disadvantaged socio‐economic groups and among men, which may be due to reduced social support for quitting, differences in self‐efficacy, poor access and adherence to treatment and targeted tobacco industry marketing [46, 47, 48]. Our results show that these differences exist throughout the age spectrum, including the age group that would be targeted by an increase in the age of sale. At the same time, an increase in the legal age of sale could exacerbate social inequalities if it promotes quit attempts and reduced uptake only among those of higher socio‐economic status and pushes those from disadvantaged backgrounds to obtain cigarettes from illicit sources. However, there was no evidence of this happening when the legal age of sale was increased from 16 to 18, with similar reductions in smoking among those eligible and not eligible for free school meals [13]. Moreover, as those aged 18–20 appear to be less dependent, it is conceivable that they would be less likely to seek out other sources of tobacco when access is reduced [49]. Previous studies suggest that laws prohibiting the sales of tobacco to minors in Europe reduce the perceived obtainability of cigarettes irrespective of socio‐economic position [50].

### Limitations

First, although this paper assessed two measures of socio‐economic status, social grade and housing tenure, which are the best predictors of smoking status after adjusting for age [27], there are several limitations with the use of these measures in young adults [51, 52]. For example, those aged 18–20 may be more likely to report living in other housing (i.e. not owned, privately rented or social housing), as they are still dependent on their parents, while social grade as an occupational measure is affected by large numbers reporting that they are still studying. Some of this confounding is reduced by within‐age‐groups comparisons. Thirdly, while the sample was designed to be representative, there is a risk of bias in terms of the characteristics of those who agree to participate. There is also a risk that respondents may fail to report their smoking status. Fourthly, it should be noted that although this study estimates that approximately 364 000 smokers would be targeted, not all are likely to go on to quit smoking and not all of those under 21 will be prevented from taking up smoking. This actual effect will, of course, depend upon how well the restrictions are implemented and whether retailers and young adults adhere to them. Future studies should attempt to model the proportion of the identified cohorts likely to change behaviour as a result of the policy. In England, age of sale laws are generally well enforced, with a fine of up to £2500 for selling to those under the age of 18; however, studies have shown that young adults often develop ways to maintain access [53]. Studies have shown an increase in successful test purchases in recent years, and this raises a concern about the effectiveness of sanctions and penalties against future behaviour [54]. Finally, this study excluded those aged 16 and 17, as the objective was to estimate the number of smokers who currently would be targeted by the increase in age of sale to 21 who were not targeted before. Increasing the age of sale to 21 could have a much bigger impact if there is an additional effect on younger teenagers, perhaps in terms of reduced access from social networks.

## Conclusion

In England, this study estimates that increasing the age of sale of cigarettes to 21 would currently target approximately 364 000 smokers who are aged 18–20. This effect would be cumulative over time by reducing uptake in future generations. Given the lower dependency in those aged 18–20 it could have a significant impact on smoking prevalence as those targeted may be less likely to seek out other illicit sources of tobacco than those in older age groups. Compared with non‐smokers aged 18–20, smokers in this age group are more likely to be from lower socio‐economic backgrounds.

## Declaration of interests

R.W. and L.S. undertake consultancy and research for and receive travel funds and hospitality from manufacturers of smoking cessation medications. E.B., J.B. and L.S. have received unrestricted research funding from Pfizer.

## Author contributions

**Emma Beard:** Conceptualization; formal analysis; methodology. **Jamie Brown:** Conceptualization; methodology. **Sarah Jackson:** Conceptualization; methodology. **Robert West:** Conceptualization; methodology. **Will Anderson:** Conceptualization; methodology. **Deborah Arnott:** Conceptualization; methodology. **Lion Shahab:** Conceptualization; methodology.

## Supporting information

**Table S1** Association between age and smoking characteristics (reference 18, 19, 20 year‐olds) restricted to data from 2019.
**Table S2** Multivariable association between smoking status (non‐smoker reference) and socio‐demographic characteristics stratified by age restricted to data from 2019.
