# Supplementary material for: Who would be targeted by increasing the legal age of sale of cigarettes from 18 to 21? A cross‐sectional study exploring the number and characteristics of smokers in England
Source: Addiction. 2021 Feb 10;116(8):2187–97. doi: 10.1111/add.15421 (PMC8436755; doi:10.1111/add.15421)
Supplement: Supplementary file 1 — Table S1 Association between age and smoking characteristics (reference 18, 19, 20 year‐olds) restricted to data from 2019. Table S2 Multivariable association between smoking status (non‐smoker reference) and socio‐demographic characteristics stratified by age restricted to data from 2019. [file ADD-116-2187-s001.docx]

**Supplementary Table 3:** Association between age and smoking characteristics (reference 18-20 year-olds) restricted to data from 2019

|  | Aged 21-24 | | | Aged 25-30 | | | Aged 31+ | | |
| --- | --- | --- | --- | --- | --- | --- | --- | --- | --- |
|  | OR | 95%CI | P | OR | 95%CI | p | OR | 95%CI | p |
| Motivation to quit | 1.82 | 0.84 to 3.96 | 0.131 | 1.73 | 0.80 to 3.75 | 0.162 | 2.10 | 1.05 to 4.19 | 0.035 |
| HIS  *Low (ref)*  *Medium*  *High* | 1.20  0.13 | 0.69 to 2.08  0.01 to 1.34 | 0.528  0.087 | 1.74  0.60 | 1.02 to 2.98  0.12 to 2.98 | 0.042  0.530 | 2.26  1.10 | 1.42 to 3.65  0.30 to 3.99 | 0.001  0.884 |
| Urges to smoke | 1.29 | 0.99 to 1.66 | 0.058 | 1.15 | 0.89 to 1.47 | 0.282 | 1.47 | 1.18 to 1.83 | <0.001 |

**Supplementary Table 4:** Multivariable association between smoking status (non-smoker reference) and socio-demographic characteristics stratified by age restricted to data from 2019

|  | Aged 18-20 | | | Aged 21-24 | | | Aged 25-30 | | | Aged 31+ | | |
| --- | --- | --- | --- | --- | --- | --- | --- | --- | --- | --- | --- | --- |
|  | OR | 95%CI | p | OR | 95%CI | P | OR | 95%CI | p | OR | 95%CI | p |
| Female | 0.72 | 0.47 to 1.10 |  | 0.73 | 0.52 to 1.02 | 0.069 | 0.78 | 0.57 to 1.07 | 0.124 | 0.83 | 0.73 to 0.94 | 0.003 |
| Social-grade  *AB (ref)*  *C1*  *C2*  *D*  *E* | 1.41  2.82  1.24  1.38 | 0.60 to 3.69  1.13 to 7.82  0.43 ti 3.77  0.46 to 4.32 |  | 0.97  1.33  2.67  1.42 | 0.52 to 1.94  0.66 to 2.81  1.32 to 5.65  0.65 to 3.22 | 0.924  0.439  0.008  0.386 | 1.24  1.88  5.39  5.57 | 0.74 to 2.15  1.09 to 3.32  3.00 to 9.60  3.00 to 10.61 | 0.420  0.026  <0.001  <0.001 | 1.50  2.13  2.61  2.96 | 1.23 to 1.83  1.73 to 2.61  2.08 to 3.26  2.35 to 3.73 | <0.001  <0.001  <0.001  <0.001 |
| Owns home | 0.68 | 0.40 to 1.15 |  | 0.44 | 0.27 to 0.71 | 0.001 | 0.68 | 0.48 to 0.96 | 0.029 | 0.31 | 0.27 to 0.36 | <0.001 |
| White | 4.69 | 2.42 to 10.26 |  | 2.41 | 1.54 to 3.92 | <0.001 | 1.71 | 1.16 to 2.56 | 0.008 | 1.52 | 1.25 to 1.85 | <0.001 |
|  | | | | | | | | | | | | |
